# Resting-state functional connectivity in multiple sclerosis patients receiving nabiximols for spasticity

**DOI:** 10.1186/s12883-023-03171-0

**Published:** 2023-03-29

**Authors:** Alberto Gajofatto, Nicolò Cardobi, Francesca Gobbin, Massimiliano Calabrese, Marco Turatti, Maria Donata Benedetti

**Affiliations:** 1 Department of Neuroscience, Biomedicine and Movement Sciences, University of Verona, Piazzale L.A. Scuro 10, Verona, 37134 Italy; 2 Unit of Neurology, Regional Multiple Sclerosis Center, Borgo Roma Hospital, Azienda Ospedaliera Universitaria Integrata, Verona, Italy

**Keywords:** Multiple sclerosis, Functional MRI, Spasticity, Cannabinoid, Nabiximols, Symptomatic therapy

## Abstract

**Background:**

Nabiximols (Sativex®) is a cannabinoid approved for multiple sclerosis (MS)-related spasticity. Its mechanism of action is partially understood, and efficacy is variable.

**Objective:**

To conduct an exploratory analysis of brain networks connectivity changes on resting state (RS) functional MRI (fMRI) of MS patients treated with nabiximols.

**Methods:**

We identified a group of MS patients treated with Sativex® at Verona University Hospital, who underwent RS brain fMRI in the 4 weeks before (T0) and 4–8 weeks after (T1) treatment start. Sativex® response was defined as ≥ 20% spasticity Numerical Rating Scale score reduction at T1 vs. T0. Connectivity changes on fMRI were compared between T0 and T1 in the whole group and according to response status. ROI-to-ROI and seed-to-voxel connectivity were evaluated.

**Results:**

Twelve MS patients (7 males) were eligible for the study. Seven patients (58.3%) resulted Sativex® responders at T1. On fMRI analysis, Sativex® exposure was associated with global brain connectivity increase (particularly in responders), decreased connectivity of motor areas, and bidirectional connectivity changes of the left cerebellum with a number of cortical areas.

**Conclusions:**

Nabiximols administration is associated with brain connectivity increase of MS patients with spasticity. Modulation of sensorimotor cortical areas and cerebellum connectivity could play a role in nabiximols effect.

## Background

Nabiximols (Delta-9-Tetrahydrocannabinol:Cannabidiol in 1:1 ratio oromucosal spray, Sativex®, Almirall) is a symptomatic drug licensed in Europe, USA and other countries as second line treatment of moderate-to-severe multiple sclerosis (MS)-related spasticity. Sativex® should be prescribed to patients already receiving one or more anti-spastic therapies (e.g. baclofen, benzodiazepines, tizanidine, and dantrolene) with suboptimal response or tolerance. In a major phase III clinical trial in MS patients, add-on treatment with nabiximols led to a clinically meaningful improvement of spasticity symptoms in 42% of cases during a run-in 4-week period, after which responders were randomized to either staying on active treatment or being switched to placebo [1]. Response to treatment was defined as a ≥ 20% reduction on the spasticity numerical rating scale (NRS) score after 4 weeks. At week 12, patients on nabiximols had a significantly lower self-reported score of spasticity severity (primary efficacy endpoint) compared to placebo.

Tetrahydrocannabinol and cannabidiol both act through interaction with CB1 and CB2 receptors that are largely distributed in several networks of the central nervous system, at the presynaptic terminal of neurons and interneurons. Although Sativex® precise mechanism of action is not completely understood, its therapeutic effect on spasticity-related symptoms may involve modulation of nociceptive as well as motor pathways since it has been shown experimentally that activation of CB1 receptors modulates excitatory synaptic transmission of dorsal horn and trigeminal nucleus neurons as well as of striatal neurons [2, 3]. Factors associated with the clinical response to Sativex® at the individual level are largely unknown, and several weeks of treatment are generally required to assess efficacy in each patient [1]. Therefore, objective predictors of therapy response would be helpful to better understand Sativex® mechanism of action and to select patients according to the likelihood of benefitting from treatment. Besides clinical and paraclinical tools, it is well established that standard brain and spinal cord MRI measures provide valuable information on central nervous system structural damage of patients with MS; however, they are unlikely to be directly associated with the extent of spasticity at the individual level. In this view, brain functional MRI (fMRI) techniques could disclose promising insights for the identification of neural networks potentially involved in the genesis and modulation of several MS symptoms, including spasticity. Previous literature has shown that brain functional networks follow complex patterns of reorganization and spatial reconfiguration in patients with MS over the disease course. Carotenuto et al. recently investigated the association of functional network topography abnormalities with disease phenotype, clinical and cognitive disability in a large group of MS patients. Resting state fMRI (RS-fMRI) was acquired cross-sectionally using degree centrality, which counts the number of functional connections of each gray matter voxel with the rest of the brain. The authors found reduced centrality in primary sensorimotor network and increased centrality in the default-mode network with abnormalities that were specific for different disease phenotypes and associated with clinical and cognitive disability [4]. A longitudinal study conducted by a different group assessed cognitive status and RS-fMRI of a large MS cohort at two time points over a period of 5 years. The authors concluded that conversion from intact cognition to impairment was associated with centrality abnormalities of both the ventral attention and the default mode networks, suggesting that progressive disruption of crucial networks occurs over time in patients with MS in parallel with clinical worsening [5].

In the present study we aimed to conduct an exploratory analysis of brain networks changes on RS-fMRI of MS patients with moderate to severe spasticity treated with Sativex® and to preliminarily investigate the effect on the connectivity of specific brain areas in responders and non-responders, comparing RS-fMRI changes before and after treatment initiation.

## Methods

### Study subjects

Participants were selected among patients with relapsing–remitting (RR) or progressive MS (2010 McDonald diagnostic criteria) [6, 7] followed at the Multiple Sclerosis Center of Verona University Hospital, Italy, who started treatment with Sativex® according to the Italian drug agency requirements between January and September 2016. Inclusion criteria were: 1) moderate to severe spasticity defined as score 4 or greater on the NRS for spasticity as reported by the patient [1]; 2) spasticity symptoms as defined above despite one or more antispastic treatment at a stable dose for the last 3 months (see also exclusion criteria); 3) brain MRI performed in the 4 weeks prior to Sativex® initiation and after 4–8 weeks from treatment start at a stable dose, according to the MRI protocol described below. Exclusion criteria were: 1) age below 18 years; 2) pregnancy; 3) clinically relevant cardiovascular and/or psychiatric disorder; 4) concomitant psychoactive substance abuse; 5) steroids for a relapse in the last 30 days; 6) any change in disease-modifying therapy, antispastic medication (including baclofen, benzodiazepines, dantrolene, tizanidine, and gabapentin), and physical therapy in the last 3 months. This study was part of a RS-fMRI research project for which the Institutional Committee of Azienda Ospedaliera Universitaria Integrata Verona approved all the experimental protocols listed in the present article. All participants gave written informed consent to undergo MRI according to institutional policy.

### Clinical evaluation

The following evaluations were done in all patients included in the study at baseline (T0, i.e. in the 4 weeks prior to Sativex® initiation) and after 4–8 weeks of continuous treatment at a stable dose (T1): 1) expanded disability status scale (EDSS) score; 2) ambulation index; 3) 25-foot walking test (if applicable); 4) spasticity NRS score. Response to treatment was defined as a ≥ 20% reduction on the NRS score at T1 compared to T0, according to previous studies showing this is the threshold for a clinically meaningful change in the spasticity burden for the patient [1].

### Functional MRI protocol

A proportion of patients followed at Verona University Hospital MS Center periodically underwent brain MRI on site with a Philips Ingenia® 1.5 Tesla scanner, as part of their disease monitoring protocol, including resting state functional MRI (RS-fMRI) sequences. For the present study, brain MRI performed at T0 and T1 in MS patients treated with Sativex® as explained above were considered for analysis.

The MRI scanner is equipped with a 15 elements head coil and foam cushions to minimize head movement. In addition, adjustable padded restraints were positioned on both sides of the head. Study subjects were instructed to remain as still as possible. RS-fMRI sequences were acquired with T2-weighted echo planar sequence (repetition time = 3000 ms, echo time = 50 ms, slice thickness = 4 mm, echo train length = 57, 30 slice, 80 dynamics, time = 240 s), in order to detect low-frequency blood-oxygen-level dependent (BOLD) fluctuations. An anatomical 3D T1-weighted spoiled gradient echo (SPGR) sequence with voxel size of 1 mm^3^ was also acquired for each subject.

### Data processing and statistical analysis

Connectivity changes on RS-fMRI were compared between T0 and T1 in the whole study group and according to treatment response status, using functional connectivity toolbox (CONN, version 17.f) [8]. All functional and anatomical sequences were first preprocessed using default CONN pipeline, which included:

- Functional realignment and unwarp (subject motion estimation and correction)
- Functional center to coordinates (translation)
- Functional slice-timing correction
- Functional outlier detection (ART-based identification of outlier scans for scrubbing)
- Functional direct segmentation and normalization (simultaneous gray/white/CSF segmentation and Montreal Neurological Institute, MNI normalization)
- Structural center to coordinates (translation)
- Structural segmentation and normalization (simultaneous gray/white/CSF segmentation and MNI normalization)
- Functional smoothing (spatial convolution with Gaussian Kernel). The smoothing was set to 8 mm.

Functional and structural volumes were resampled at 2 mm.

Band pass filtering was also performed, removing temporal frequencies below 0.008 Hz or above 0.09 Hz from BOLD signal to focus on slow-frequency fluctuations while minimizing the influence of physiological, head-motion and other noise sources. The quality check of the denoise procedure was performed visually using quality assurance report plots generated by CONN itself.

After pre-processing, a second level group analysis step was applied. Region of interest (ROI)-to-ROI and seed-to-voxel connectivity were evaluated, using uncorrected statistically significant threshold of *p* < 0.01 and false discovery rate (FDR)-corrected statistical significance threshold of *p* < 0.05. ROI-to-ROI connectivity were carried out on all 164 ROIs included in CONN. Among them, 132 ROIs were atlas of cortical and subcortical areas from the fMRI of the brain software library (FSL) Harvard–Oxford atlas, as well as cerebellar areas from the automated anatomical labelling (AAL) atlas; 32 ROIs were atlases of resting state networks (RSN) represented by default mode network (DMN), sensorimotor network, visual networks, salience networks, dorsal attention networks, frontoparietal networks, language networks, cerebellar networks. RSN and ROIs were each independently analyzed; functional connectivity pairs were obtained with 164 ROIs, in which all of the RSN ROIs and atlas ROIs were combined. Only the significant ROI-to-ROI connection were selected, according to the threshold above mentioned. ROI-to-ROI connectivity were visually elaborated as connectome ring maps with associated tables reporting the statistically significant ROI-to-ROI effect and corresponding *p* value (both uncorrected and FDR corrected).

Seed-to-voxel connectivity was carried out selecting left and right precentral and postcentral gyrus as a seed ROI. The significant clusters were also reported in tables with MNI coordinates and size. Uncorrected, FDR-corrected and Family-Wise Error (FWE) *p*-values were also reported.

Clinical data were reported as frequencies for categorical variables and as median with minimum–maximum range for quantitative variables. Between groups comparisons were performed using the Fisher exact test for categorical variables and the Mann–Whitney test for quantitative variables, setting the statistical significance at *p* < 0.05. The statistical tests were performed using SPSS 25 (IBM Corp).

### Statistical power calculation

No preliminary data exist for RS-fMRI changes induced by Sativex® in MS patients. In a previous RS-fMRI study on the effect of tetra-hydro-cannabinol in 12 healthy volunteers, authors were able to identify significant changes in the activation of some brain areas, particularly involving the sensorimotor network [9]. Accordingly, we assumed for our study that in order to detect a signal change on fMRI of approximately 0.5% and a spatial smoothing of 5 mm, at least 12 patients were needed to insure a 80% power with alfa = 0.05 at the single voxel level [10].

## Results

Between January and September 2016 18 MS patients started treatment with Sativex® at our Institution. Of these, 12 (7 males and 5 females) were eligible for the study according to inclusion/exclusion criteria. All patients were on a stable physical rehabilitation program with a similar protocol at our Institution. At T0 median age was 51 years (range, 36—73), disease duration 21.5 years (10–37), EDSS score 6.0 (4.5–8.0), and NRS score for spasticity 8 (5–9). The clinical course was relapsing–remitting in 2 and secondary progressive in 10 patients. At T1 7 patients (58.3%) were classified as Sativex® responders, based on NRS score reduction ≥ 20% at T1 compared to T0. Clinical characteristics of patients are summarized in Table 1.

**Table 1:** Main clinical characteristics of study patients according to Sativex® response status

	**All patients (*n* = 12)**	**Responders (*n* = 7)**	**Non-responders (*n* = 5)**	*P*-value*
**age at T0**; years, median (range)	51 (36—73)	50 (43—54)	62 (36—73)	*p* = 0.13
**male gender**; N(%)	7 (58%)	6 (86%)	1	n.a
**disease duration at T0**; years, median (range)	21.5 (10—37)	22 (12—37)	21 (10—33)	*p* = 0.91
**EDSS score at T0**; median (range)	6.0 (4.5—8.0)	6.5 (5.0—8.0)	5.5 (4.5—7)	*p* = 0.34
**spasticity NRS at T0**; median (range)	8 (5—9)	7 (5—9)	8 (8- 9)	*p* = 0.12
**spasticity NRS at T1**; median (range)	6.5 (4—8)	5 (4—7)	7 (7—8)	
**∆NRS T1-T0**; %, median (range)	21% (0—33%)	25% (20%—33%)	12.5% (0—12.5%)	

*Abbreviations: EDSS* Expanded disability status scale, *NRS* Numerical rating scale

* Responders vs. non-responders comparison (see details in the Methods section)

**Fig. 1:**
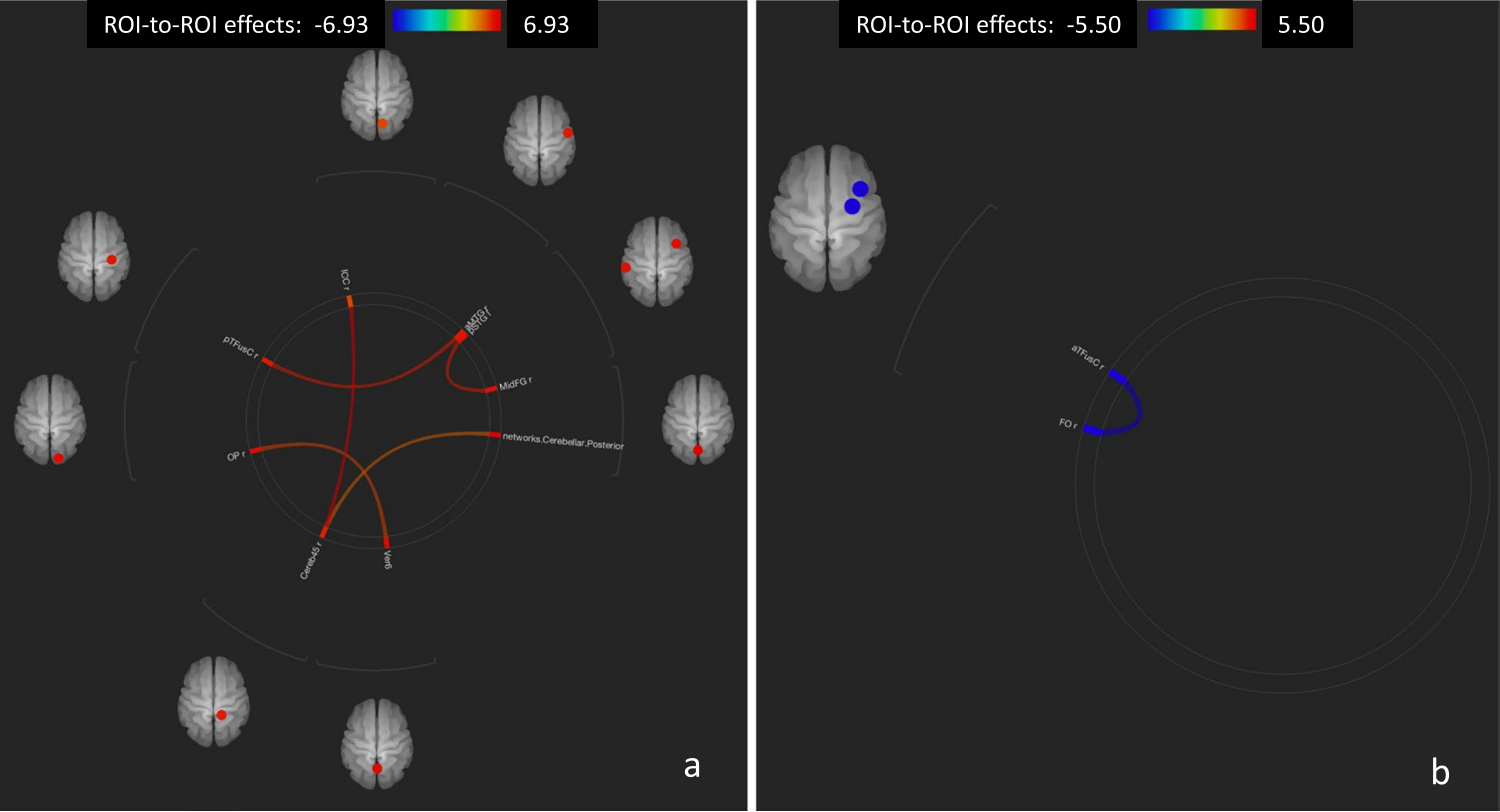
Region of interest (ROI)-to-ROI connectome ring maps of all selected ROI seeds in Post vs. Pre nabiximols exposure (all patients). Increased connectivity (**a**). Decreased connectivity (**b**). The colour links are obtained at a ROI-to-ROI connections height threshold (FDR) of *P* < 0.05, seed ROI height threshold (uncorrected) of *P* < 0.05, with one-sided positive seed level correction and permutation tests. The colour bar indicates the statistical T value. Abbreviations: ROI (region of interest), FDR (false discovery rate), ICC l (Intracalcarine Cortex left), aMTG r (Middle Temporal Gyrus, anterior division Right), pSTG l (Superior Temporal Gyrus, posterior division Left), MidFG r (Middle Frontal Gyrus Right), Ver6 (Vermis 6), Cereb45 r (Cerebellum 4 5 Right), OP r (Occipital Pole Right), pTFusC r (Temporal Fusiform Cortex, posterior division Right), aTFusC r (Temporal Fusiform Cortex, anterior division Right), FO r (Frontal Operculum Cortex Right)

RS-fMRI ROI-to-ROI analysis in all study subjects at T1 compared to T0 showed a significant association between ongoing Sativex® therapy and global brain connectivity increase, decreased connectivity of motor areas, and bidirectional connectivity modulation of the left cerebellum with a number of cortical areas, particularly the left pre-central and post-central gyrus bilaterally (Table 2 and Fig. 1).

**Table 2:** Post vs. Pre-nabiximols exposure contrast in region-of-interest connections (all patients)

Analysis Unit	Statistic	p-unc	p-FDR
Cereb45 r -ICC r	T(9) = 6.93	0.0000	0.0057
ICC r -Cereb45 r	T(9) = 6.93	0.0000	0.0057
MidFG r -pSTG l	T(9) = 6.17	0.0001	0.0137
pSTG l -MidFG r	T(9) = 6.17	0.0001	0.0137
pTFusC r -aMTG r	T(9) = 6.12	0.0001	0.0145
aMTG r -pTFusC r	T(9) = 6.12	0.0001	0.0145
Ver6 -OP r	T(9) = 5.30	0.0002	0.0412
OP r -Ver6	T(9) = 5.30	0.0002	0.0412
Cereb45 r -(165)	T(9) = 4.72	0.0005	0.0454

Region-of-interest (ROI) connections with threshold ROI to ROI connection by intensity at a 0.05 one-sided (positive) FDR-*P* value and threshold seed ROIs at a 0.05 uncorrected *P* value

*Abbreviations: FDR* False discovery rate, *l* left, *r* right, *Cereb45* Cerebellum 4 5, *ICC* Intracalcarine Cortex, *MidFG* Middle Frontal Gyrus, *pSTG* Superior Temporal Gyrus posterior division, *pTFusC* Temporal Fusiform Cortex posterior division, *aMTG* Middle Temporal Gyrus anterior division, *Ver6* vermis 6, *OP* Occipital Pole; 165, networks.Cerebellar.Posterior

**Fig. 2:**
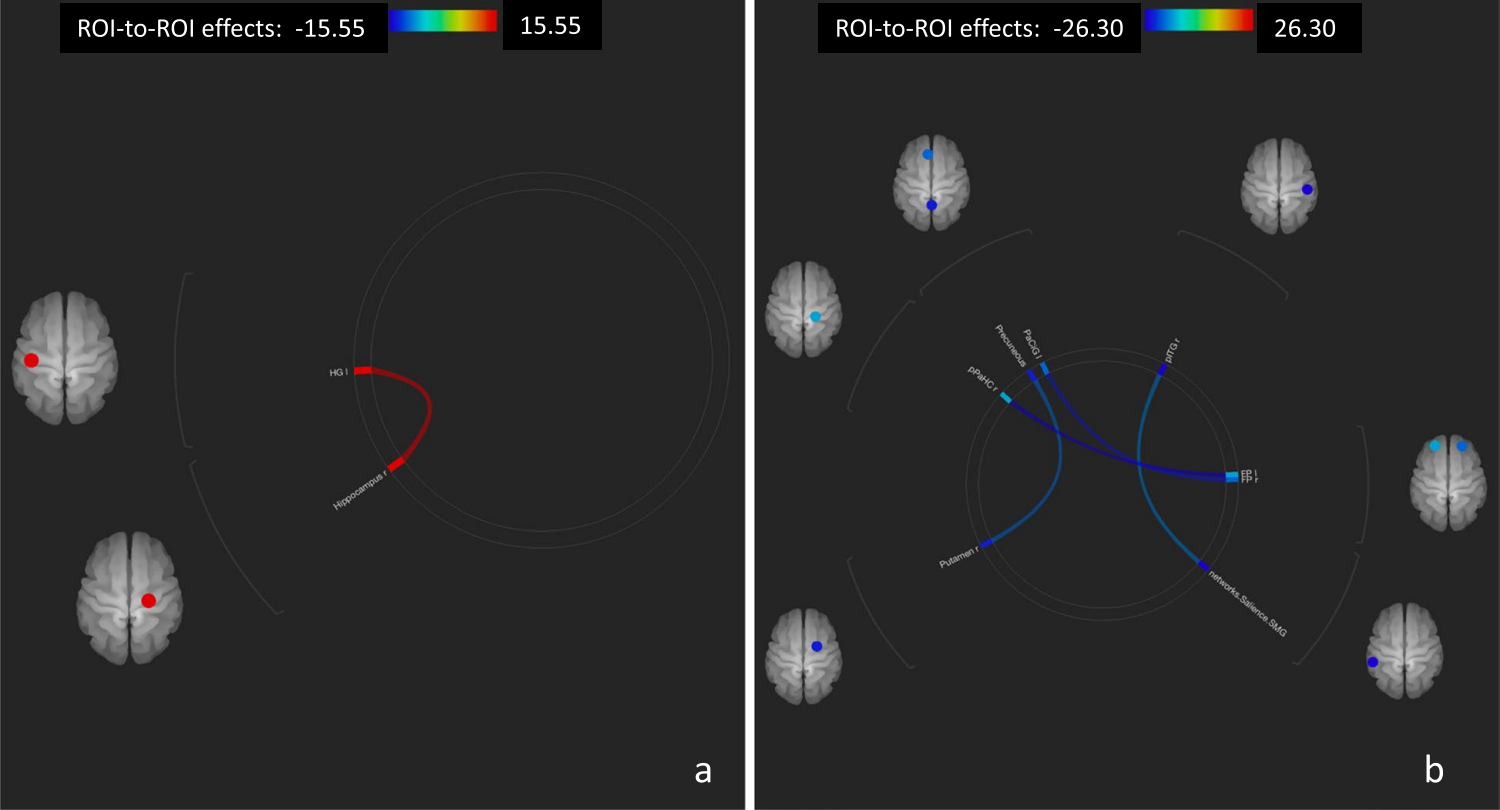
ROI-to-ROI connectome ring maps, Post- vs. Pre- condition, Sativex® responders. Increased connectivity (**a**). Decreased connectivity (**b**). The colour links are obtained at a ROI-to-ROI connections height threshold (FDR) of *P* < 0.05, seed ROI height threshold (uncorrected) of *P* < 0.05, with one-sided positive seed level correction and permutation tests. The colour bar indicates the statistical T value. HG l (Heschl's Gyrus Left), pPaHC r (Parahippocampal Gyrus, posterior division Right), PaCiG l (Paracingulate Gyrus Left), pITG r (Inferior Temporal Gyrus, posterior division Right), FP r (Frontal Pole Right), FP l (Frontal Pole Left)

**Fig. 3:**
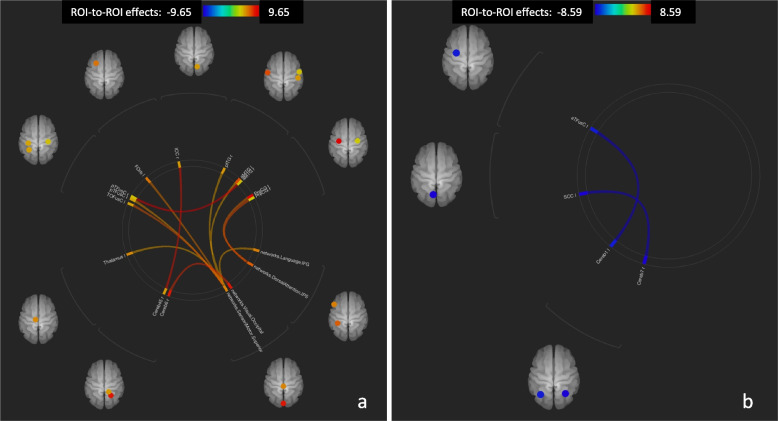
ROI-to-ROI connectome ring maps, Post- vs. Pre- condition, Sativex® non-responders. Increased connectivity (**a**). Decreased connectivity (**b**). The colour links are obtained at a ROI-to-ROI connections height threshold (FDR) of *P* < 0.05, seed ROI height threshold (uncorrected) of *P* < 0.05, with one-sided positive seed level correction and permutation tests. The colour bar indicates the statistical T value. Cereb45 r (Cerebellum 4 5 Right), Cereb6 r (Cerebellum 6 Right), TOFusC l (Temporal Occipital Fusiform Cortex Left), pTFusC r (Temporal Fusiform Cortex, posterior division Right), pTFusC l (Temporal Fusiform Cortex, posterior division Left), FOrb l (Frontal Orbital Cortex Left), ICC r (Intracalcarine Cortex Right), pITG r (Inferior Temporal Gyrus, posterior division Right), aMTG r (Middle Temporal Gyrus, anterior division Right), aMTG l (Middle Temporal Gyrus, anterior division Left), PreCG r (Precentral Gyrus Right), PreCG l (Precentral Gyrus Left), Cereb1 l (Cerebellum Crus1 Left), Cereb7 r (Cerebellum 7b Right), SCC l (Supracalcarine Cortex Left), aTFusC l (Temporal Fusiform Cortex, anterior division Left)

The global growth of connection observed at T1 vs. T0 was more evident in responders (Fig. 2), while there was a more pronounced reduction of motor areas connectivity in non-responders compared to responders (Fig. 3).

Of note, ROI-to-ROI analysis showed that global brain connectivity at T0 was lower in non-responders compared to responders. This difference was still present, although attenuated, at T1, i.e. during Sativex® treatment.

**Fig. 4:**
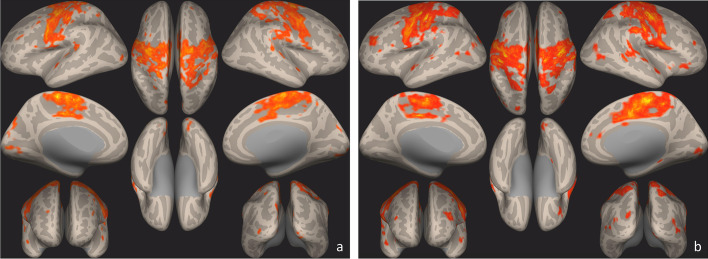
Seed-to-Voxel statistically significant clusters maps using precentral and postcentral gyrus as a seed ROIs, all patients. Pre- (**a**) and Post- (**b**) Sativex exposure. Results are visualized as clusters on inflated brain (*p* < 0.05, FDR corrected). In post-condition (**b**), there is an increase in connectivity in pre and post central gyrus compared to the pre-condition (**a**)

**Fig. 5:**
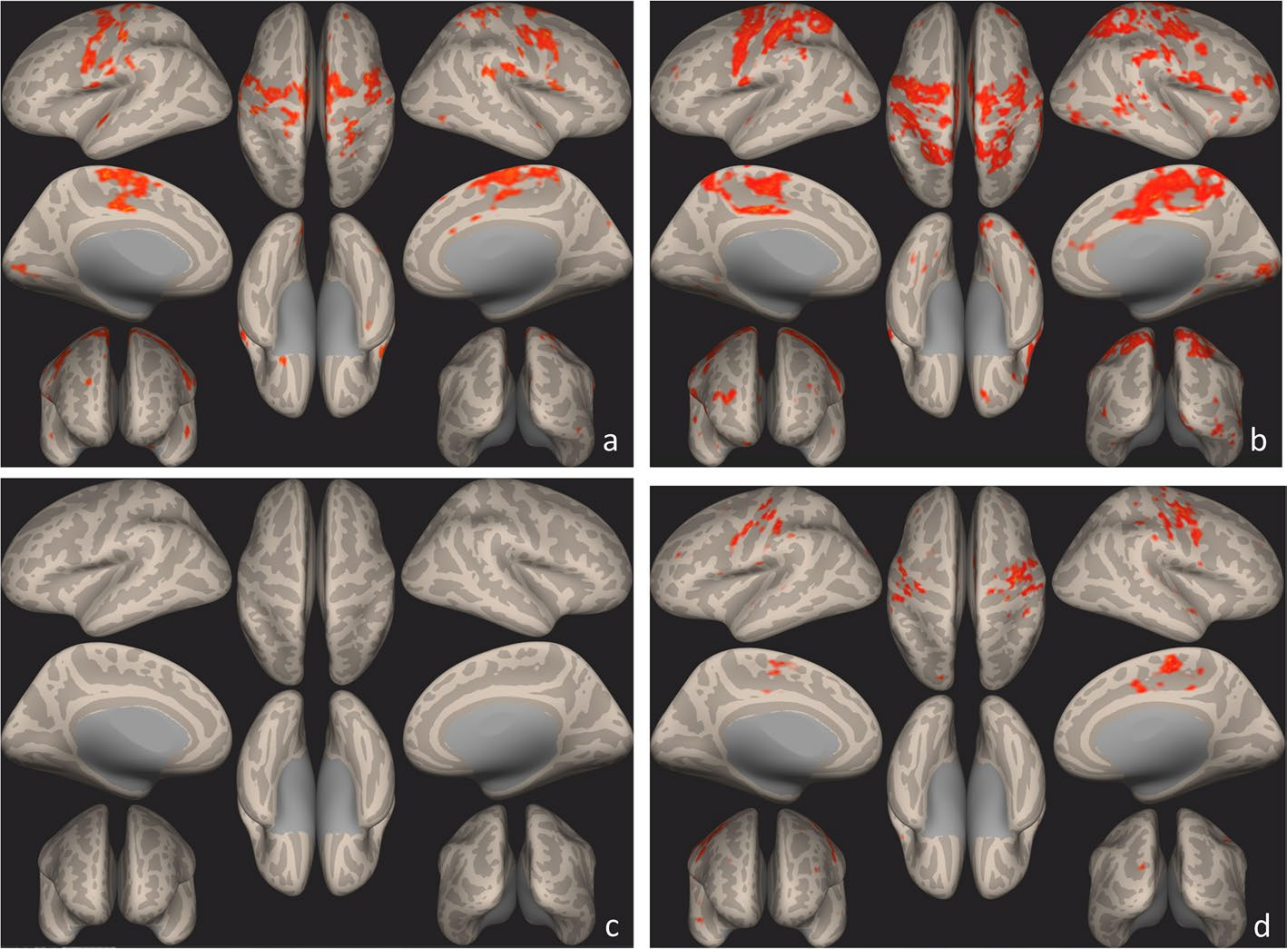
Seed-to-Voxel statistically significant clusters maps using precentral and postcentral gyrus as a seed ROIs, according to responder status. Pre (**a**) and Post (**b**) condition in Sativex® non responders and Pre (**c**) and Post (**d**) condition in responders. Results are visualized as clusters on inflated brain (*p* < 0.05, FDR corrected). In post condition (**b**), there is an increase in connectivity in pre and post central gyrus compared to the pre-condition in non-responders. Same condition is evident in responders (**c**, **d**), despite in pre-condition (**c**) there are no significant clusters

Seed–to–voxel analysis on the whole study group and on subgroups according to treatment response at T1 compared to T0 showed a significant connectivity increase of left pre-central and post-central gyri with several ipsilateral and contralateral cortical areas of the brain (Table 3; Figs. 4 and 5).

**Table 3:** Seed-to-Voxel statistically significant clusters with coordinates in Montreal Neurological Institute space (pre-condition, all patients)

Clusters (x,y,z)	size	size p-FWE	size p-FDR	size p-unc	peak p-FWE	peak p-unc
-06 -28 + 60	13,562	0.000000	0.000000	0.000000	0.017673	0.000000
-12 -78 + 28	169	0.000000	0.000000	0.000000	0.999737	0.000024
-12 -88 + 00	146	0.000000	0.000000	0.000000	0.985643	0.000010
+ 18 -80 -16	128	0.000001	0.000000	0.000000	1.000000	0.000061
+ 34 + 48 + 32	93	0.000037	0.000013	0.000000	1.000000	0.000053
-24 -52 + 50	56	0.002689	0.000788	0.000023	0.978046	0.000009
-36 -82 -02	45	0.011395	0.002875	0.000098	0.985474	0.000010
+ 08 -80 + 30	28	0.130618	0.030721	0.001199	1.000000	0.000196
-44 + 38 + 28	26	0.175995	0.037766	0.001658	1.000000	0.000106

*Abbreviations: FDR* False discovery rate, *FWE* Family-Wise Error, *unc* uncorrected

## Discussion

In the present study we conducted an exploratory analysis of brain connectivity by RS-fMRI in patients with MS treated with Sativex with the primary aim of comparing brain networks activation before and after treatment initiation. At a single University MS Center, we enrolled a group of 12 patients predominantly with progressive MS and moderate-to-severe motor disability eligible for Sativex therapy in line with the typical characteristics of MS patients treated with this drug [11].

Overall, Sativex treatment was associated with global brain connectivity increase (particularly in responders), decreased connectivity of motor areas, and bidirectional connectivity modulation of the left cerebellum with a number of cortical areas, particularly the left pre-central and post-central gyrus bilaterally, compared to pre-treatment status. These findings could be linked to the interaction of THC and CBD with CB1 – and to a lesser extent CB2 – receptors, which are known to modulate synaptic activity of neurons involved in nociceptive and motor pathways [2, 3]. In addition, connectivity modulation was observed in brain regions in which there is expression of CB1 receptor, particularly the pre-central and post-central gyri according to JuSpace tool (https://github.com/juryxy/JuSpace/tree/JuSpace_v1.4/JuSpace_v1.4/PETatlas) and to Terry et al [12].

Available data on the changes induced by cannabinoids use in patients with MS as measured by fMRI are scarce. It has been suggested that smoked cannabis can induce structural brain MRI changes and worsen cognitive functioning in MS patients [13]. A recent study investigated the effect of cannabinoid withdrawal on cognition and on both structural and functional brain MRI measures in a group of 19 MS patients who were long term cannabis users in comparison to a group of 20 MS patients continuing cannabis administration [14]. The authors reported that cannabinoid use interruption was associated with an improvement in cognition and with significantly increased task-related fMRI activation in brain regions known to be associated with cognitive functioning, i.e. bilateral inferior frontal gyri, caudate and declive/cerebellum.

In a different clinical context, a RS-fMRI study conducted in patients with schizophrenia and co-occurring cannabis use disorder reported that low dose THC increased basal brain connectivity of the default mode network (DMN), which involves anterior cingulate cortex, precuneus, medial prefrontal cortex, and angular gyrus connections and is related to global cognitive functioning [15].

Several studies on MS cases with fatigue and/or neuropsychological impairment, which frequently coexist to spasticity, have shown an increased maladaptive DMN connectivity, suggesting the involvement of network reorganization processes in the clinical expression of MS symptoms [4, 5, 16–18]. Keeping in mind the results of our study, it could be speculated that Sativex therapeutic effect, in addition to motor areas connectivity decrease, might be also mediated by modulation of non-sensorimotor networks, such as cortical-cerebellum connections, which could determine beneficial effects on symptoms related to spasticity.

RS-fMRI in MS patients have generally reported an increased global brain connectivity compared to healthy subjects as a result of CNS plasticity mechanisms overcoming functional failure of networks involved by the disease processes [19]. In addition, neural plasticity is more efficient in the early compared to the advanced stages of MS: as progression of structural damage proceeds compensatory mechanisms may exhaust after a definite functional reserve threshold has been reached [20]. In this view, our findings showing an increased connectivity in responders to Sativex compared to non-responders could be interpreted as the result of greater residual CNS functional reserve available to obtain a treatment effect as opposed to compensatory mechanisms exhaustion leading to no benefit with Sativex administration. Based on these findings, it could be hypothesized that RS fMRI is a potential predictive biomarker of nabiximols therapeutic effect in MS-related spasticity. These results need to be interpreted very cautiously considering that the study sample size was calculated for whole group analysis and statistical power was reduced for comparisons between responders and non-responders subgroups. In our study, non-responders were numerically older and had a higher NRS score for spasticity at T0 compared to responders (Table 1), suggesting that the first subgroup could have more advanced disease than the second one. In addition, male sex was more frequent in responders that in non-responders. Although the study was not sufficiently powered for these comparisons, the differences were not statistically significant. Nevertheless, it may not be excluded that differences in brain connectivity observed between responders and non-responders at T0 were the reflection of the above-mentioned patient characteristics (i.e. sex, age and spasticity score) rather than an independent predictor of response to Sativex.

Our study is limited by the observational design, small sample size, baseline differences between subgroups, and lack of a control group; however, our findings expand the knowledge in the field and provide preliminary data to be confirmed in future research. Longitudinal fMRI studies on larger cohorts of MS patients are needed to better characterize clinical correlates of cannabinoid therapeutic effects that could help the management of spasticity and other symptoms.

## Conclusions

Treatment with Sativex® appears to increase overall brain connectivity of MS patients with spasticity, particularly in responders. In addition, therapeutic response to Sativex® is associated with greater pre-treatment global brain connectivity compared to suboptimal response. Finally, modulation of motor areas and cerebellum connectivity could play a role in the clinical effect of Sativex®.

## Data Availability

The datasets analysed for the present research are available upon appropriate request to the corresponding author.
